# A New Synthetic Pathway for the Bioproduction of Glycolic Acid From Lignocellulosic Sugars Aimed at Maximal Carbon Conservation

**DOI:** 10.3389/fbioe.2019.00359

**Published:** 2019-11-27

**Authors:** Cléa Lachaux, Cláudio J. R. Frazao, Franziska Krauβer, Nicolas Morin, Thomas Walther, Jean Marie François

**Affiliations:** ^1^Toulouse Biotechnology Institute (TBI), Université de Toulouse, CNRS, INRA, INSA, Toulouse, France; ^2^TWB, Toulouse, France

**Keywords:** synthetic biology, metabolic engineering, glycolic acid, aldolase, white biotechnology

## Abstract

Glycolic acid is a two-carbon α-hydroxy acid with many applications in industrial sectors including packaging, fine chemistry, cosmetics, and pharmaceutics. Currently, glycolic acid is chemically manufactured from fossil resources. This chemical mode of production is raising some concerns regarding its use in health for personal care. Microbial production of GA stands as a remarkable challenge to meet these concerns, while responding to the increasing demand to produce bio-sourced products from renewable carbon resources. We here report on the design and expression of a novel non-natural pathway of glycolic acid in *E. coli*. The originality of this new pathway, termed “glycoptimus” relies on two pillars. On the one hand, it requires the overexpression of three naturally occurring *E. coli* genes, namely *kdsD* encoding a D-arabinose-5-P isomerase, *fsaA* encoding a class 1 aldolase that cleaves D-arabinose-5-P into glyceraldehyde-3-P and glycolaldehyde, and *aldA* coding for an aldehyde dehydrogenase that oxidizes glycoladehyde in glycolate. These three genes constitute the “glycoptimus module.” On the other hand, the expression of these genes together with a reshaping of the central carbon metabolism should enable a production of glycolic acid from pentose and hexose at a molar ratio of 2.5 and 3, respectively, which corresponds to 50% increase as compared to the existing pathways. We demonstrated the ‘*in vivo*’ potentiality of this pathway using an *E. coli* strain, which constitutively expressed the glycoptimus module and whose carbon flow in glycolysis was blocked at the level of glyceraldehyde-3-P dehydrogenase reaction step. This engineered strain was cultivated on a permissive medium containing malate and D-glucose. Upon exhaustion of malate, addition of either D-glucose, D-xylose or L-arabinose led to the production of glycolic acid reaching about 30% of the maximum molar yield. Further improvements at the level of enzymes, strains and bioprocess engineering are awaited to increase yield and titer, rendering the microbial production of glycolic acid affordable for a cost-effective industrial process.

## Introduction

Glycolic acid (GA) is a two-carbon α-hydroxy acid (HOCH_2_COOH) with dual properties of both alcohol and moderately acid (pKa 3.83). It is the simplest organic acid, which finds multiple applications in the cosmetic industry to improve skin texture, in pharmaceutic industries to treat skin diseases, in textile industry as a dyeing and tanning agent, in food industry as flavor and preservative as well as for cleaning and sanitizer agent in household and industry (https://www.grandviewresearch.com/industry-analysis/glycolic-acid-industry). Polymerization of glycolic acid alone or with other acids monomer such as lactic acid yields thermoplastic resins with excellent gas barrier properties. These polymers have the capability of being hydrolysed in aqueous environments gradually and controllably, making them good candidates for packaging materials or dissolvable sutures useful for biomedical applications (Fredenberg et al., 2011; Gädda et al., 2014). This large panel of glycolic acid applications accounts for the fact that the demand for this organic acid is constantly growing from US$ ~300 million in 2017 to US$ ~406 million in 2023, exhibiting a CAGR of 6.83% during this forecast period (https://www.researchandmarkets.com/reports/4542547/glycolic-acid-market-forecasts-from-2018-to-2023). Even though glycolic acid can be extracted from plants such as sugarcane, pineapple, and sugar beets, it is chiefly chemically manufactured from fossil resources by carbonylation of formaldehyde at high-pressure temperature (Drent et al., 2001). Alternatively, it can be produced from the enzymatic conversion of glycolonitrile using microbial nitrilases (He et al., 2010) or by bioconversion of ethylene glycol using *Gluconobacter oxydans* as the biocatalyst (Kataoka et al., 2001; Wei et al., 2009). However, these chemo-enzymatic methods rely on known irritant and carcinogenic chemicals, making their use problematic for some applications, especially within the personal care products industry.

**Figure 1:**
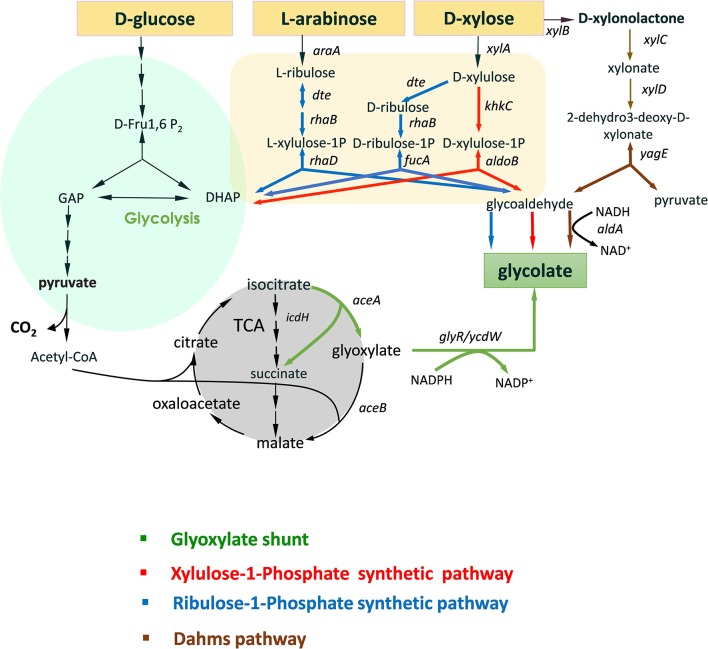
Scheme of natural and non-natural pathways for glycolic acid production from D-glucose, D-xylose, and L-arabinose. Only the most significant genes (enzymes) are illustrated. Legend: *araA (*arabinose isomerase*); dte* (tagatose 6-P epimerase); *rhaB* (L-rhamnulokinase); *rhaD* (L-rhamno 1-phosphate aldolase); *xylA* (xylose isomerase); *fucA* (L-fuculose-1-phosphate aldolase); *khKC* (keto-hexokinase/fructose-1phosphate kinase); *aldoB* (aldolase-B); *xylB* (xylose dehydrogenase; *xylC* (xylonolactone lactonase); *xylD* (xylonate dehydratase); *yagE* (KDX aldolase); *aldA* (glycoaldehyde dehydrogenase).

In addition to these health concerns, another strong impetus to develop alternative and sustainable solutions for glycolic acid production comes from environmental and societal needs to reduce our dependence on fossil-based products and to promote bio-production from renewable carbon sources using microbial cell factories (Aguilar et al., 2013). GA represents a good opportunity for such an advancement, motivated by the fact that there are no natural microbial producers to produce at high yield this platform molecule from sugars (Salusjarvi et al., 2019). Consequently, biotechnological production of this simple organic acid from renewable resources has received a substantial interest in the recent years leading to the engineering of four different routes as depicted in Figure 1 (and reviewed in Salusjarvi et al., 2019). The glyoxylate shunt (GS) is the natural pathway, the physiological function of which is to bypass the oxidative decarboxylation of TCA cycle, thereby conserving carbon skeletons for biomass (Dolan and Welch, 2018). This bypass starts at the level of isocitrate, which is aldolytically cleaved into succinate and glyoxylate by isocitrate lyase (ICL) encoded by *aceA* in *E. coli*. The first attempt for the bioproduction of glycolic acid from D-glucose via the glyoxylate shunt in *Escherichia coli* has been patented by METEX (Soucaille, 2007). To achieve a production of *ca* 57 g/L at 45% of the theoretical yield, 13 genetic modifications have been implemented, including the overexpression of the NADPH glyoxylate reductase (GLR) encoded by *ycdW*/*ghrA*, attenuation of isocitrate dehydrogenase (IDH) and deletion of side pathways that prevent lactate and acetate production as well as to the oxidation of glycolate. Further genetic modifications brought about by Deng et al. (2018) resulted in an engineered strain able to produce 65 g/L at 90% of the theoretical yield. D-xylose and ethanol have been used as carbon source for GA production via engineering of GS pathway in *Saccharomyces cerevisiae* and *Kluyveromyces lactis* (Koivistoinen et al., 2013), whereas acetate was the carbon substrate for glycolate production by a engineered *C. glutamicum* strain for TCA cycle and GS pathway and using D-glucose for growth (Zahoor et al., 2014). However, the exploitation of GS for glycolic production presents at least two major limitations. The first one deals with the fact that the pathway has been optimized to use only D-glucose as the carbon source, either by releasing glucose repression of the glyoxylate shunt genes in *E.coli* (Gui et al., 1996) or by expressing these genes under non-repressible glucose promoter in the yeast *S. cerevisiae* (Koivistoinen et al., 2013). The second problem is related to the NADPH preference of the glyoxylate reductase enzymes, which generates redox imbalance in the cell. In *E. coli*, this problem is even reinforced if ICDH activity is attenuated since it results in a reduction of NADPH availability.

Three -non-natural- GA pathways (Figure 1) have been constructed and expressed in bacteria and yeast that could in part overcome the problems stated above. Firstly, the production of GA by these routes bypasses the central carbon metabolism. As such, it requires fewer enzymatic reaction steps than with the natural GS: only three enzymatic steps are required for the X1P pathway (Cam et al., 2016) while four for the R1P (Pereira et al., 2016a) and for the Dahms pathway (Cabulong et al., 2018). Lowering the number of reaction steps might have a positive effect on the production yield as it reduces the energy cost for protein synthesis and metabolic burden and reduce loss of intermediates by competitive pathways (Bilgin and Wagner, 2012). Second, GA is produced from glycolaldehyde by a NAD^+^-dependent (glycol)aldehyde dehydrogenase. However, only pentose sugars can be assimilated by these non-natural pathways. In addition to this limitation, only two carbons of the C5-intermediates are utilized to produce GA whereas the three remained carbons are diverted into biomass via DHAP. To overcome in part this carbon loss, X1P and R1P pathways have been combined with GS bypass, enabling assimilation of both hexose and pentose sugars (Alkim et al., 2016; Pereira et al., 2016b). Conversion of C5 sugar to GA with high yield has been also proposed by linking part of the GS pathway with two reverse glyoxylate pathway (RGP) enzymes malate thiokinase and malyl-coA lyase through Dahms pathway to recuperate pyruvate and recycle malate into glyoxylate (Cabulong et al., 2018).

The combination of the natural glyoxylate shunt with non-natural GA producing pathways is very promising, as for instance, a conversion rate of D-xylose into GA at 92% of the theoretical yield was obtained by the Dahms-GS-RGP (Cabulong et al., 2018). However, these pathways still present some metabolic hurdles such as redox imbalance between NADH and NADPH. In addition, they do not reach the maximum energy yield (Y^E^) which is the maximum amount of product that can be formed from a substrate (Dugar and Stephanopoulos, 2011). This Y^E^ value is pathway independent and is determined by the ratio γ_s_/γ_p_ where γ_s_ and γ_p_ are the reduction degrees of the substrate and the product, respectively. Accordingly, it can be calculated that Y^E^ of glycolic acid (γ_p_ = 6) from D-glucose (γ_S_ = 24) and from pentose (γ_S_ = 20) would be 4 and 3.3, respectively. However, this yield can only be reached if the biological system is capable of uptaking a carbon mole as CO_2_. Alternatively, if the loss of CO_2_ at the level of pyruvate is prevented (see Figure 1), the theoretical yield of GA from D-glucose and pentose would be 3 and 2.5 moles/mole, which is 50% higher than that obtained by natural and non-natural pathways (Table 1). Given this postulate, the purpose of this communication was to design and validate a novel -non-natural- GA pathway enabling an efficient assimilation of pentose and hexose derived from lignocellulosic biomass into GA to reach these yields and that in the meantime solving most of the problems raised by the existing ones and notably to overcome redox imbalance.

**Table 1:** Maximal yield of glycolic acid as calculated from stoichiometric and thermodynamic rules.

**Pathway**	**Yield of GA (mole/mole)**		**References**
	**D-xylose/L-arabinose**	**D-glucose**	
Glyoxylate shunt (GS) pathway	1.66	2	Deng et al., 2018
Ribulose-1P pathway	1	0	Pereira et al., 2016a
Xylulose-1P pathway	1	0	Cam et al., 2016
Ribulose-1P+ GS pathways	2	2	Pereira et al., 2016a
Xylulose-1P + GS pathways	2	2	Alkim et al., 2016
Dahms-GS-RGP pathway	2	–	Cabulong et al., 2018
Glycoptimus pathway	2.5	3	This work

## Materials and Methods

### Chemicals and Reagents

All chemicals and solvents were purchased from Sigma-Aldrich unless otherwise stated. Restriction endonucleases and DNA-modifying enzymes were from New England Biolabs. DNA plasmid were extracted using GeneJET Plasmid Miniprep Kit (Thermo Scientific). DNA sequencing was subcontracted to Eurofins SAS (Ebersberg, Germany).

### Plasmid Construction

Vectors pZA23, pZA33, pZE23, and pZS23 from Expressys^®^ were used as they are inducible by IPTG and harbor a lightened structure of the *lacI* gene which reduces its size (2 358 to 3 764 bp) and they are modulable (easy to change replication origin, resistance marker and vector promoter by restriction/ligation). In this study, the promoter PA1lac0-1 in pZA33 has been replaced by the constitutive promoter proC and by the inducible promoter P_tac_, generating pZA37 and pZA36, respectively. Likewise, the promoter PA1lac0-1 of pZS23 has been replaced by proD generating pZS28.

Gene cloning was carried out using NEBuilder HIFI DNA Assembly Master Mix (NEB E2621). This method enables several fragments to be assembled in a single step. The commercial mixture provided by New England Biolabs contains (a) an exonuclease, which creates 3′ single strand ends, which facilitates assembly of the fragments, which share a sequence complementarity; (b) a polymerase, which fills the empty spaces after the fragments have been assembled; and (c) a ligase, which links fragments together. The *E. coli kdsD, fsaA* and *aldA* genes were amplified from the genome DNA extracted from *E. coli* K12 MG1655 by PCR using primers described in Table S1. Fragments (*kdsD* + *fsaA or aldA*) were then inserted by HiFi assembly^®^ into linearized beforehand pZ with primers hybridizing on either side of the MCS. All the plasmids have been verified by sequencing. The resulting plasmids bearing the *kdsD, fsaA, and aldA* genes are reported In Table 2.

**Table 2:** Plasmids used or constructed in this study.

**Name**	**Description**	**Source**
pET28a	Kan ^R^, ori ColE1, Ptac	Novagen
pZA33	Cm^R^, ori p15A, P_a1lac0−1_	Expressys
pZA27	Kan ^R^, ori p15A, proC	This study
pZA36	Cm ^R^, ori p15A, P_tac_	This study
pZA38	Cm ^R^, ori p15A, proC	This study
pZS23	Kan ^R^, ori pSC101, P_a1lac0−1_	Expressys
pZS27	Kan ^R^, ori pSC101, proD	This study
pA4	pZS23 aldA	This study
pA7	pZS27 aldA	This study
pKF3	pZA36 kdsD fsaA	This study
pKF6	pZS38 aldA	This study
pVT-FSAA	pET28 fsaA	This study
pVT-KDSD	pET28 kdsD	This study
pVT-ALDA	pET28 aldA	This study

### Strain Construction and Transformation

The *E. coli* strains used in this work are listed in Table 3. Gene deletion (i.e., *glcD, fucA, mgsA,pfkA, ptsG*) was made by transduction using the phage P1vir. The preparation of the lysates P1vir and the transduction procedures were carried out as described in Bremer et al. (1984) with slight modifications. Strains (donor strain) from KEIO collection (Murakami et al., 2007) bearing a single deletion and a kanamycin antibiotic-resistance cassette was inoculated (200 μl of an overnight preculture made in LB) in 5 ml of LB containing 0.2% D-glucose and 5 mM CaCl_2_ for 30 min at 37°C. Then, 100 μl of P1vir lysate (~ 5 × 10^8^ phages/ml) was added to each donor culture and incubated at 37°C for 2 to 3 h until the culture was clear and the cells were completely lysed (Baba et al., 2006). The lysates were recovered by filtration using 25 mm sterile syringe filters with a 0.2 μm support membrane (Pall) and preserved at 4°C. To delete the gene of interest, the receiving strain was infected with P1vir bearing the donor gene deletion cassette having a kanamycin resistance. For this purpose, the receiving strain was previously cultivated in 5 ml LB medium at 37°C, collected by centrifugation at 1 500 g for 10 min and re-suspended in 1.5 ml of 10 mM MgSO_4_ and 5 mM CaCl_2_. P1vir Lysate bearing the gene deletion cassette from the donor strain was added (0.1 ml) to the receiving strain suspension and incubated for 30 min at 37°C. Then, 0.1 ml of 1 M sodium citrate was added, then 1 mL LB, and this cellular suspension was incubated of 1 h at 37°C, 200 rpm before being spread on a solid LB medium with the appropriate antibiotic. Colonies were screened by PCR to isolate successful transduction events. Removal of the antibiotic cassette was carried out by transformed of the bacteria strains with pCP20 plasmid bearing the FLP recombinase, followed by PCR checking.

**Table 3:** *E. coli* strains used and constructed in this work.

**Strain**	**Genotype**	**References**
MG1655	F- λ- *ilvG*- *rfb*-50 *rph*-1	ATCC 407076
NEB5	*fhuA2 Δ(argF-lacZ)U169 phoA glnV44 Φ80 Δ(lacZ)M15 gyrA96 recA1 relA1 endA1 thi-1 hsdR17*	NEB
BL21 (DE3)	*huA2 [lon] ompT gal (λ DE3) [dcm] ΔhsdS)*	NEB
Screen00	MG1655 ΔtktA ΔtktB ΔglcD containing pZA36	This study
Screen09	Screen00 containing pZA36 kdsD fsaA (pKF3) and pZS23 aldA (pA4)	This study
Screen14	MG1655 ΔtktA ΔtktB ΔglcD containing pZA27	This study
Screen23	Screen00 containing pZA37 kdsD fsaA (pKF6) and pZA28aldA (pA7)	This study
WC3G gapA-	W3CG *F^−^, LAM^−^, gapA 10::Tn10, IN(rrnD-rrnE), rph^−1^*	Ganter and Pluckthun, 1990
BW25113	F-, *Δ(araD-araB)567, ΔlacZ4787*(::rrnB-3), *λ^−^, rph-1, Δ(rhaD-rhaB)568, hsdR514*	Baba et al., 2006
JW2771	BW25113 ΔfucA	Baba et al., 2006
JW4364	BW25113 ΔarcA	Baba et al., 2006
JW5129	BW25113 ΔmgsA	Baba et al., 2006
JW2946	BW25113 ΔglcD	Baba et al., 2006
JW2771	BW25113 ΔfucA	Baba et al., 2006
JW3887	BW25113 ΔpfkA	Baba et al., 2006
Glyco00	WC3G *gapA-10::Tn10* ΔglcD ΔarcA ΔmgsA ΔfucA ΔpfkA galP^ProD^	This study
Glyco09	GA00 containing pZA36 kdsD fsaA (pKF3) and pZS23 aldA (pA4)	This study
Glyco23	GA00 containing pZA38 kdsD fsaA (pKF6) and pZA27 aldA (pA7)	This study

The competent non-commercial strains were prepared according to the protocol of Chung et al. (1989) with minor modifications as followed. A pre-culture was made overnight in LB overnight. Fresh LB culture was then inoculate with cells at a DO_600_ of 0.1. When DO_600_ reaches about 0.5, 2 ml of culture was collected and to the resulting pellet was resuspended in 300 μl TSS buffer [2.5%_(w/v)_ PEG 3350, 1 M MgCl_2_, 5%_(vol/vol)_ DMSO]. After 10 min on ice, plasmid of interest was added to the cell suspension, which was further incubated for 30 min on ice. This step was followed by a heat shock at 42°C for 90 s. The transformed cells were put on ice for 10 min then 400 μl LB was added and the culture was incubated at 200 rpm for 1 h at 30°C. After centrifugation at 8 000 rpm for 2.5 min, the cell pellet was resuspended in 600 μl of LB and 150 μl were spread on a solid LB plates with the appropriate antibiotic.

### Cultures Conditions

For molecular biology techniques, the bacteria strains were cultured in the LB medium (10 g/L trypton, 5 g/L yeast extracts and 5 g/L NaCl), and 5 g/L agar was added for solid medium in petri dishes. For GA production, the mineral medium M9 was used. Unless otherwise stated, it contained per liter: 18 g Na_2_${\text{HPO}}_{4}^{*}$12H_2_O, 3 g KH_2_PO_4_, 0.5 g NaCl, 2 g NH_4_Cl, 0.5 g ${\text{MgSO}}_{4}^{*}$7H_2_O, 0.015 ${\text{CaCl}}_{2}^{*}$2H_2_O, 1 ml of 0.06 M FeCl_3_ from a 1000 × stock solution in concentrated HCl, 2 ml of 10 mM thiamine HCl stock solution, 20 g MOPS, and 1 ml of trace element solution (1000 × solution of 0.5 g Na_2_EDTA^*^2H_2_O, 0.18 g ${\text{CoCl}}_{2}^{*}$6H_2_O, 0.18 g ${\text{ZnSO}}_{4}^{*}$7H_2_O, 0.4 g Na_2_${\text{MoO}}_{4}^{*}$2H_2_O, 0.1 g H_3_BO_3_, 0.12 g ${\text{MnSO}}_{4}^{*}$H_2_O, 0.12 g CuCl_2_ × H_2_O). The carbon source D-glucose, L-arabinose or D-xylose was added at a final concentration of 10 g/l, the pH was adjusted to 7 with acid 3-(N-morpholino) propanesulphonic (MOPS) and then filter sterilized through 0.2 μm membranes. The antibiotics ampicillin, kanamycin and chloramphenicol were added when required at concentrations of 100, 50, and 25 mg/L, respectively. For the *E. coli* strains defective in transketolase activity *(*Δ*tktA* Δ*tktB*), M9 medium was supplemented with 500 μM L-phenylalanine, 250 μM L-tyrosine, 200 μM L-tryptophan, 6 μM p-aminobenzoate, 6 μM p-hydroxydenzoate, and 280 μM shikimate and trace of LB (20% _V/V_). For the strains defective in glyceraldehyde-3-phosphate dehydrogenase activity (Δ*gapA*), the M9 medium was completed with 0.4 g/L malic acid adjusted at pH 7 with KOH. When required, the appropriate antibiotics was added to the medium at 100 μg/mL for ampicillin, 50 μg/mL for kanamycin, or 25 μg/mL for chloramphenicol. The bacteria cultures were placed in rotatory shaker at 200 rpm and at 37°C. Growth was monitored by measuring absorbance at 600 nm with a spectrophotometer (Biochrom Libra S11).

### Enzymes Production, Purification, and Assays

The *kdsD, fsaA*, and *aldA* genes were amplified using primers in Table S1 and cloned in the expression vector pET28a (Novagen). The *E. coli* strain BL21 (DE3) transformed with the plasmid bearing these genes were inoculated from a pre-culture made in LB-kanamycin (50 mg/ L) in 200 mL of LB-Kanamycin at 600 nm (DO_600_) of 0.1 at 16°C in a rotary shaker at 200 rpm. When DO_600_ reached 0.6–0.8, the expression of the protein of interest was induced by addition of IPTG at 1 mM final concentration 1 mM IPTG. After 16 h at 16°C, the culture was collected by centrifugation at 4,800 rpm for 15 min at 4°C. The cell pellet was re-suspended in 1.5 mL washing buffer (50 mM HEPES, pH 7.5; 0.3 M NaCl) and sonicated four times (30 s each) at 30% power on ice. The HIS-tagged proteins were purified using cobalt resin according to the protocol described in commercial kit (Clontech). Purification of the protein was verified by SDS-PAGE electrophoresis and protein concentration was measured by Bradford method (Bradford, 1976)

Enzymes assays were made in a Tris-Cl, 100 mM pH 7.5/10 mM MgCl_2_ buffer at 37°C. Unless otherwise stated, KdsD activity was measured in a coupled assay in the presence of 3 mM NAD^+^ and 5 mM D-Ribu-5P with 10 μg/ml of each of FsaA and AldA. FSA was measured by coupling GAP produced from cleavage of 3 mM Ara5P with the oxidation of 0.2 mM NADH at 340 nm in the presence of 1 U/ml of triose isomerase and glycerol-3-P dehydrogenase. AldA was measured in the same buffer by reduction of NAD^+^ (3 mM) at 340 nm in the presence of 5 mM glycolaldehyde.

### Analytical Methods

Extracellular metabolites were determined by high performance liquid chromatography (HPLC) with an Ultimate 3000 chromatograph (Dionex, Sunnyvale, USA). The HPLC system was equipped with a cation exchange column (Aminex, HPX87H; 300 × 7.8 mm, 9 μm, BioRad), an automatic injector (WPS-3000RS, Dionex), an IR detector (RID 10A, Shimadzu) and a UV detector (SPD-20A, Shimadzu). The sample injection volume was 20 μL, and the compounds were separated in an Aminex HPX-87H column protected by a Micro-Guard Cation H pre-column (BioRad, USA). The separation was performed at 35°C with 1.25 mM H_2_SO_4_ at 0.5 mL min^−1^ as mobile phase. All samples were centrifuged (2 min at 10,000 g) and syringe-filtered (0.2 μm), and the resulting supernatant kept at −20°C until analysis.

### Genome-Scale Modeling and Flux Balance Analysis

The iJO1366 genome-scale *E. coli* model (Orth et al., 2011) has been adapted to simulate GA production through the glycoptimus pathway. Notably, a modified *fsaA* reaction was added to the model to allow the conversion of arabinose-5P into glyceraldehyde-3P and glycolaldehyde. Parsimonious flux balance analyses (pFBA) were performed using the OptFlux software (Rocha et al., 2010), using either D-glucose, L-arabinose or D-xylose as a carbon source. For each simulation, the uptake rate of the carbon source was arbitrarily set at 10 mmol.${\text{g}}_{\text{CDW}}^{-1}$.h^−1^. Non-growth associated maintenance was set at 3.15 mmol_ATP_.${\text{g}}_{\text{CDW}}^{-1}$.h^−1^, and reactions involved in the electron transport system were constrained to simulate a realistic P/O ratio, as previously described (Orth et al., 2011). pFBA simulations were performed using GA export as the objective function to maximize.

### Statistical Methods

Statistical analyses were conducted in Microsoft Excel^®^ using the Analysis ToolPak package. A two-tailed unpaired *t*-test was to use to compare fluorescence induction levels, in which an alpha level of *p* < 0.05 was set for significance.

## Results and Discussion

### Pathway Design and Modeling

**Figure 2:**
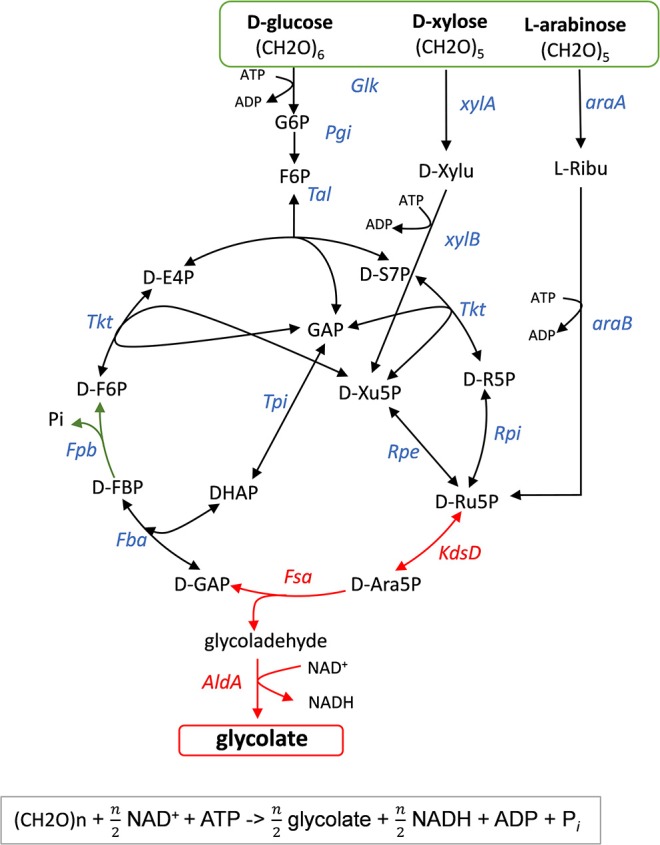
Scheme of the glycoptimus pathway. Key steps are D-arabinose-5-phosphate isomerase (KdsD) and fructose-6-phosphate aldolase (FsaA) shown as red arrows. In green is shown the oxidation of glycoladehyde to glycolate by glycoladehyde dehydrogenase AldA. Glk, glucokinase; Pgi, phosphoglucoseiomserase, Tal, transaldolase; Tkt, transketolase; Rpi, ribose-5-P isomerase; Rpe, ribulose-5-P epimerase; Fba, fructose1,6-bisphosphate aldolase; Tpi, triose isomerase; Fbp, fructose1,6-bisphosphate phosphatase; xylA, xylose isomerase; xyB, xylulose 5-P kinase; araA, arabinose isomerase; araB, L-ribulose-5-P kinase. Stoichiometry of the glycoptimus reaction with sugar is given below.

The pathway design that could maximally convert the lignocellulose-derived sugars D-glucose, D-xylose and L-arabinose into GA with, in theory, no loss of CO_2_ is illustrated in Figure 2. This route relies on two key enzymatic steps that are sequentially catalyzed by an isomerase encoded by *kdsD*/*yrbH* (Meredith and Woodard, 2003), which interconverts D-Ribu-5P and D-Ara-5P, and an aldolase encoded by *fsaA* (Schurmann and Sprenger, 2001) that cleaves this intermediate into glycolaldehyde and GAP. The latter is shuttled back to D-Ribu-5P via the pentose phosphate pathway. Overall, this cyclic pathway should in theory produces 3 moles GA per mole of consumed D-glucose and 2.5 moles GA per mole of consumed pentose sugar, as confirmed by parsimonious flux balance analyses (Table S2 and Figure S1). The proposed pathway is thermodynamically favorable as deduced from the sum of Δ_r_G'^0^ of each reaction using Equilibrator (http://equilibrator.weizmann.ac.il/) (Figure S2). The large negative Gibbs energy results from the phosphorylation of the sugar and the oxidation of glycolaldehyde by the glycoladehyde dehydrogenase encoded by *aldA* (Figure 2). In addition, this metabolic pathway which we termed “glycoptimus” is redox balanced since NADH produced can be regenerated to NAD^+^ via oxidative phosphorylation, leading to ATP production. This energy provision is sufficient to sustain GA production, based on the assumption that (i) 2 moles ATP are produced per mole of NADH and (ii) 2 moles of ATP are required for the active transport and phosphorylation of each mole of sugar. As flux balance analyses equilibrate redox and energetic balances, modeling of the glycoptimus pathway highlighted that optimal flux toward GA production should be accompanied with a reduction of the P/O ratio. Indeed, the model adjusts the production of ATP to the needs of the system (i.e., sugar uptake and non-growth associated maintenance). Excess of redox cofactors is therefore equilibrated by the introduction of futile cycles within the model. These results highlight the fact that an *in vivo* strategy toward a maximal GA production yield might require further process development (e.g., use of micro-aerobic conditions) to adapt oxidative phosphorylation and redox balance during the production phase. Alternatively, the introduction of additional reactions to oxidize the excess of NADH produced by AldA could be considered.

### Kinetic Properties of Key Enzyme Encoded by *kdsD* and *fsaA* and *in vitro* Validation

Our rational to design this pathway arose from the fact that *E. coli* genome is endowed with *kdsD*/*yrbH* gene that encodes an Ara5P isomerase (Meredith and Woodard, 2003). The physiological function of this enzyme is to generate D-Ara-5P from D-Ribu-5P as the first precursor in the biosynthesis of 3-deoxy-(D)-manno-octulosonate (KDO), a sugar moiety located in the lipopolysaccharide layer of most Gram-negative bacteria. We have revised the kinetic properties of the three key enzymes in the proposed pathway, namely KdsD, FsaA, and AldA, all being expressed in *E. coli* BL21 (DE3) strain as HIS-tagged fusion proteins. The enzyme KdsD was confirmed to be highly specific toward D-Ara-5P and D-Ribu-5P. However, the determined catalytic constant (k_cat_) and Michaelis-Menten affinity constant (K_M_) values for the D-Ribu-5P substrate were found to be distinct of those previously reported by Meredith and Woodard (2003). In particular, a four-fold higher K_M_ (D-Ribu-5P, ~1.3 mM) and a 10-fold lower k_cat_ (D-Ribu-5P, 24 s^−1^) were obtained in our study (Table 4). These differences could be ascribed to the presence of the HIS-tag at the N-terminus of the protein, which could interfere with the catalytic activity of this enzyme. For the aldolytic cleavage of D-Ara-5P into glycolaldehyde et GAP, the aldolase encoded by *fsaA* was retained based on the work of Garrabou et al. (2009) which reported a higher specificity of this enzyme to D-Ara-5P than to F6P; the latter being cleaved into dihydroxyacetone and GAP (Schurmann and Sprenger, 2001). While the physiological function of this class I aldolase in *E. coli* is still unclear, this enzyme is profitably employed as a biocatalyst for stereoselective C-C bond formation (Clapes et al., 2010). The K_M_ and k_cat_ determined on D-Ara-5P using purified His-tagged FsaA enzyme (named FsaA-HIS) was in the range of those obtained by Garrabou et al. (2009) (Table 4). Since KdsD and FsaA both catalyse reversible reactions, the feasibility of this pathway is ensured by coupling the NAD^+^-dependent glycolaldehyde dehydrogenase to the oxidative phosphorylation chain (resulting in NAD^+^ regeneration coupled to ATP synthesis). We thus determined the kinetic properties of the purified AldA (Table 4). While the K_M_ for glycoladehyde was similar to that reported in the original work of Caballero et al. (1983), the k_cat_ was 10-fold lower, which may be due to difference in enzyme purification and condition of enzymatic assay. Nonetheless, determination of catalytic efficiencies (expressed as k_cat_/K_M_) indicated that FsaA is most likely the rate-limiting step in the metabolic route yielding GA from D-Ribu5-P (Table 4).

**Table 4:** Catalytic constants of arabinose-5P isomerase (KDSD), fructose-6P aldolase (FSA), aldehyde dehydrogenase (AldA) purified from *E. coli* expressing the protein flanked of a His-tag at the N-terminus of the sequence.

**Enzyme**	**Substrate**	**K_**M**_ (mM)**	**V_**max**_ (μmoles.min^**−1**^ mg^**−1**^)**	**k_**cat**_ (s^**−1**^)**	**k_**cat**_/K_**M**_ (s^**−1**^.mM^**−1**^)**
KdsD-His	D-ribulose-5P	1.30 ± 0.12	1.4 ± 0.17	24	18.4
FsaA-His	D-arabinose-5P	0.65 ± 0.15	0.26 ± 0.09	3.6	5.5
AldA-His	glycolaldehyde	0.21 ± 0.05	1.66 ± 0.31	52	247.5

**Figure 3:**
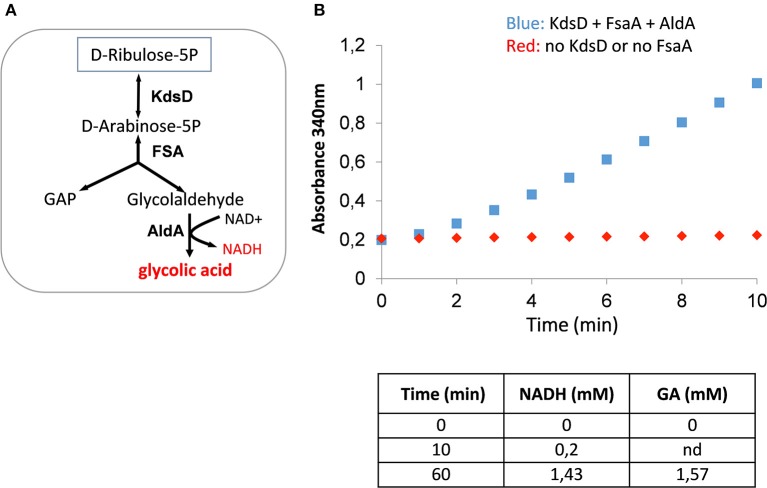
*In vitro* validation of the KdsD-FsaA pathway for GA production. In **(A)** *In vitro* enzyme assay scheme for conversion of D-ribulose 5-phosphate into GA. In **(B)** is shown the NADH production resulting from the conversion of D-ribulose 5-phosphate into GA. The reaction was made in 1 ml buffer solution (100 mM Tris-HCl pH 7.5, 10 mM Mgcl_2_, 0.1 mM MnCL_2_, and 1 mM sodium thiamine pyrophosphate) in the presence of 5 mM D-ribulose 5-phosphate, 2 mM NAD^+^, and 10 μg of each of purified KdsD, FsaA, and AldA enzyme.

We then validated the functioning of the glycoptimus route *in vitro* by incubating D-Ribu5-P with purified KdsD, FsaA, and AldA (Figure 3). Pathway validity was assessed by following the increase of absorbance at 340 nm corresponding to NADH production coupled with the stoichiometric formation of GA as confirmed by HPLC analyses. In the absence of either KdsD or FsaA, no NADH formation was observed as expected.

### Growth-Based Screening of the Glycoptimus Pathway

The most direct way to show that a synthetic pathway works *in vivo* is to express the genes of that pathway in a plasmid under the dependence of an inducible promoter. Upon promoter induction with a suitable inducer, the production of the compound of interest can be monitored. However, it is also wiser to associate the implementation of a synthetic pathway with a phenotypical trait that is fully dependent of the *in vivo* functioning of the pathway. With this procedure, effects of plasmids copy number, promoter strength, RBS, position of the genes in the operon can be investigated in a combinatorial manner to find out the expression modules leading to the best production pathway. With respect to our glycoptimus pathway, we took advantage of the previous finding that an *E. coli* strain defective in transketolase encoded by *tktA* and *tktB* is unable to grow on D-xylose or L-arabinose (Josephson and Fraenkel, 1969, 1974). Expression of *kdsD-fsaA-aldA* module pathway shall restore cell growth in the aforementioned sugar substrates. With this approach (see Figure 2), the assimilation of D-xylose or L-arabinose will result in the formation of glycoladehyde and GAP. While the former can be oxidized into GA, GAP will be used for cell growth (see Figure S3). The herein proposed screening strategy shall in principle meet two criteria: (i) growth rescue of a Δ*tktA* Δ*tktB* mutant on D-xylose (or L- arabinose), and (ii) GA production correlated to growth proficiency of the mutant strain bearing the pathway. To avoid degradation of GA product, the *glcD* gene encoding the glycolate oxidase (Pellicer et al., 1996) has been also deleted from the template screening strain.

**Figure 4:**
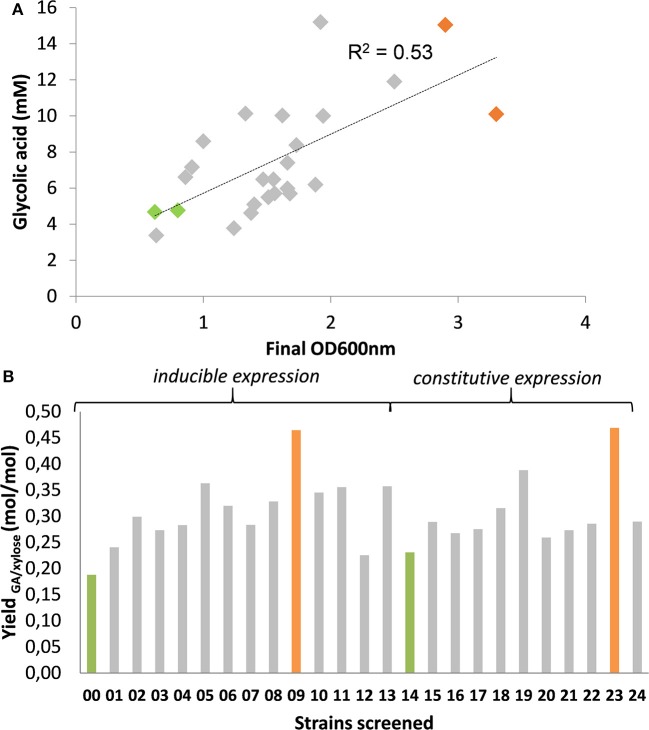
*In vivo* screen of strains for production of GA expressing *KdsD-fsaA* + *aldA* operon. Strain MG1655 Δ*tktA* Δ*tktB* Δ*glcD* was transformed with pZS bearing various *kdsD-fsaA* + *aldA* operons as described in Table S3, Figure S4 and cultivated on M9 supplemented in the presence of 1% (w:v) D-xylose. After an adaptation phase of about 16–24 h, cells were centrifuged (4,000 rpm, 5 min) and re-inoculated at DO_600_ of 0.5 in 50 mL of the same medium in 250 ml baffled shake flask. Growth was monitored at 600 nm **(A)** and after 150 h, when OD_600_ was stable, which is after 150 h, sample was taken to measure GA in the supernatant. **(B)** Results shown are the mean of two independent experiments.

Accordingly, we initially constructed a single *kdsD-fsaA-aldA* operon under the control of the IPTG-inducible P_lac_ promoter expressed from medium- and high-copy number plasmids from the pZ collection. Unexpectedly, none of these constructs rescued growth of the MG1655Δ*tktA* Δ*tktB* Δ*glcD* on D-xylose. Upon sequencing of these plasmids, we systematically found a deletion in the P_*lac*_ promotor, which was due to a repeat motif of this promotor present in these pZ plasmids. This event has been recognized earlier and explained by a replication-slipping mechanism, which often occurs due to the presence of short repeat motifs in these plasmids (Kawe et al., 2009). Replacement of P_lac_ by P_tac_ was also unsuccessful, resulting instead in Tn10 transposon insertion in the *kdsD* gene. We therefore changed our strategy by expressing *aldA* separately from *kdsD-fsaA*. We found that splitting the operon in two different but compatible plasmids enabled growth rescue of the Δ*tktA* Δ*tktB* Δ*glcD* strain. Encouraged by this observation, we constructed 24 different expression systems in which combination of 3 types of vectors carrying three different but compatible replication origin and 4 different promoters (2 inducible P_tac_ and P_lac_ and 2 constitutive proC and prod, Davis et al., 2011) were attempted, while genes order of *kdsD-fsaA* and their original RBS sequences remained unchanged. These 24 different expression systems (see details on plasmid constructs for this purpose in Table S3 and Figure S4) were transformed into MG1655Δ*tktA* Δ*tktB* Δ*glcD* strain. After an overnight pre-culture in M9 medium with 10 g/l of D-glucose supplemented with 0.1% (w/v) LB and 0.1% (w/v) yeast extract, the cells were collected by centrifugation, washed once with sterile water and resuspended at an initial 0D_600_ of 0.5 in M9 containing 10 g/L D-xylose complemented with the necessary auxotrophic requirements. After 46 h of cell cultivation during which growth was monitored (notice that growth rate of the transformants was rather low in the range of 0.03 to 0.05 h^−1^, data not shown), the supernatant was collected to measure GA produced and D-xylose consumed by HPLC. The results supported in part our assertion as the amount of GA produced was roughly correlated (*R*^2^ ~ 0. 53) with growth (Figure 4A). A slightly better correlation was obtained between GA produced and D-xylose consumed (*R*^2^ ~ 0.72, see Figure S5), which may indicate that GA was produced in part independently to growth. Also, it was unanticipated to find a significant production of GA (4.7 mM) in strains bearing empty plasmids (namely strain Screen00 and Screen14), which otherwise hardly grew on D-xylose. A careful analysis of the exometabolome in these transformants showed the presence of D-xylulose whose accumulation was the highest in the control strains Screen00 and Screen14. Moreover, an inverse correlation could be drawn by plotting GA yield per D-xylose vs. D-xylulose yield per D-xylose (see Figure S5B). While these data support the notion that GA production is coupled to growth, it still does not explain where GA comes from in the non-growing strains. There is at least two possibilities, which are likely additive. On the one hand, GA could arise from the aldolytic cleavage of D-xylulose by FsaA. We indeed found a weak activity of this enzyme on D-xylulose, which exhibited a very weak affinity on this substrate (Figure S6). Alternatively or complementary to this possibility, part of the hyperaccumulated phosphorylated sugars that are found in the non-growing strain (up to 15 mM, data not shown) can be diverted to GA using the endogenous activity of KdsD, FsaA, or its homolog FsaB and AldA (data no shown).

**Figure 5:**
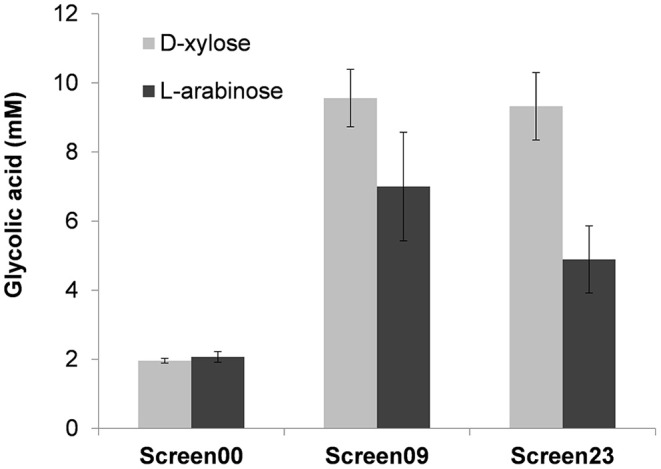
Glycolic acid production in MG1655 Δ*tktA* Δ*tktB* Δ*glcD* on D-xylose and L-arabinose. Strain Screen09 was MG1655 Δ*tktA* Δ*tktB* Δ*glcD* transformed with the IPTG inducible operon *kdsD-fsaA* (pKF3 in pZA36) and pA4 (aldA in pZS23), whereas strain Screen23 was MG1655 Δ*tktA* Δ*tktB* Δ*glcD* transformed with pKF6 (*kdsD-fsaA* in pZA38 under constitutive promoter proC) and pA7 (*aldA* in pZS27 under constitutive promoter proD). The strains were cultivated in mineral medium M9 at 37°C in the presence of D-xylose or L-arabinose at 10 g/L. GA was determined after 46 h of growth by HPLC. The results shown are the mean ± SD of three independent experiments.

From the correlation of GA produced to D-xylose consumed, it turned out that strains termed Screen09 and Screen23 were the best producers with a product yield of 0.48 mol GA/mol of D-xylose (Figure 4B). Interestingly, in both strains, the *kdsD-fsaA* operon and *aldA* gene were carried out in a medium- and a low-copy plasmid, respectively. While the expression system of these genes was IPTG inducible in strain Screen09, *kdsD-fsaA*, and *aldA* were driven by the constitutive promoter proD and proC, respectively. We also validated that these two strains could produce GA from L-arabinose. As shown in Figure 5, the production of GA on D-xylose and L-arabinose was significantly better in strain Screen09 and Screen23 than in the control strain Screen00, which showed that the module pathway driven either by an IPTG inducible (strain Screen09) or by constitutive promoters (strain Screen23) was operational. In addition, although the titer of GA on D-xylose was higher than on L-arabinose, the yield was actually comparable (i.e., 0.5 mole / mole of sugar), indicating that D-xylose was better assimilated than L-arabinose.

### Further Engineering of the Central Carbon Metabolism and Effect on GA Production

While the results reported above proved that the *kdsD-fsaA* + *aldA* module pathway is functional *in vivo*, we wanted to go one-step further by refactoring the central carbon metabolism to drive more carbon for GA production. Therefore, our starting *E. coli* strain was W3CG, which is defective in the lower part of glycolysis due to a *Tn10* transposon insertion into *gapA* encoding glyceraldehyde-3P dehydrogenase (Ganter and Pluckthun, 1990). The homologous gene *gapB* was found to be inactive in this strain (Tsuruno et al., 2015). Hence, this strain requires a gluconeogenic substrate such as malate in addition to D-glucose to grow. Our metabolic engineering for maximal GA production implied to delete competing but non-essential pathways for growth, as they could siphon intermediates out of the glycoptimus pathway (genetic intervention are schematically depicted in Figure S7). Therefore, the oxidation of glycolate into glyoxylate was abrogated by deletion of *glcD* encoding glycolate dehydrogenase (Pellicer et al., 1996). Meanwhile, *arcA*, which encodes the DNA-transcriptional regulator of the two-component system *ArcAB* implicated in redox state signaling (Malpica et al., 2006) was disrupted since it was reported to repress *aldA* (Pellicer et al., 1999). Deletion of *mgsA* encoding methylglyoxal synthase (Saadat and Harrison, 1998) and *fucA* encoding a fuculose aldolase was also carried out in the recipient strain to reduce siphoning of DHAP/GAP out of the glycoptimus pathway. We also deleted *pfkA* encoding PFK-1 isoform I, which contributes to 90% of the phosphofructokinase activity (Kotlarz et al., 1975) to avoid wasteful recycling of F6P into F1,6P_2_. Finally, the sugar/H^+^ transporter encoded by *galP* was overexpressed by swapping its own promoter by the strong constitutive proD promoter to favor uptake of D-glucose and D-xylose (Henderson, 1990).

**Figure 6:**
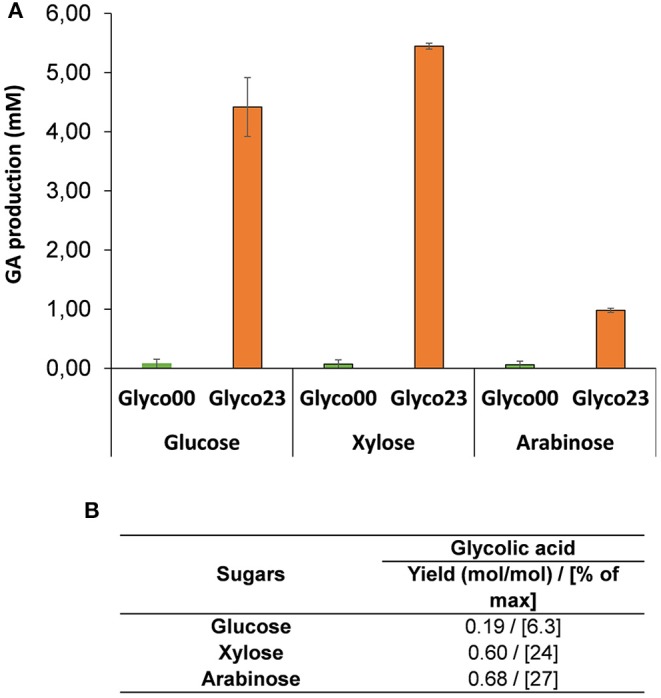
Titer and yield of GA in strain Glyco and Glyco23 on D-xylose and L-arabinose. Strain Glyco00 corresponded to *E. coli* WC3G Δ*gapA* Δ*glcD* Δ*arcA* Δ*mgsA* Δ*fucA* Δ*pkf proD-galP*, transformed with empty vector pZA36 and pZS27 whereas pKF6 and pA7, was used to yield Glyco23 strain. The strains were preliminary cultivated in 50 ml of mineral medium M9 containing 5 g/L malate and 1 g/L D-xylose at 37°C in 250 ml shake flask at 37°C. When malate was completely consumed, 5 g/L of either D-xylose or L-arabinose was added and the culture was further incubated for 24 h. **(A)** Titer and **(B)** yield were determined after 24 of cultures. The results are the mean ± SD of two independent experiments.

This engineered strain termed Glyco00 was transformed with the constitutive expression system 23 corresponding to plasmid pKF6 (*kdsD-fsaA* under proC promoter in a medium copy plasmid) and pA7 (*aldA* under proD promoter in a low copy plasmid) to yield strain Glyco23. Both the untransformed and transformed strains were firstly cultivated in mineral medium M9 in the presence of malate (5 g/L) and D-xylose (1 g/L), with a growth rate in the range of 0.07 −0.11 h^−1^. After complete consumption of malate, D-xylose, L-arabinose or D-glucose were added at 5 g/L. GA production was monitored for 24 h. It can be seen in Figure 6 that strain Glyco23 was able to produce GA from hexose and pentose sugars. However, the production yield was only 0.2, 0.6, and 0.68 mole GA per mole of D-glucose, D-xylose and L-arabinose, respectively (Figure 6B). This very low yield is not in accordance with our model. In addition, only a small amount of sugar has been consumed during the 48 h-incubation period, suggesting that the expression of the glycoptimus pathway has impaired the energetic status of the cell. Ongoing work is underway to clarify this problem. It is interesting to notice that the control strain Glyco00 did not accumulate GA, which supports the hypothesis that the production of GA seen in screen00 (see Figure 4) might originate from D-xylulose and/or phosphorylated sugars that were accumulated, what did not occur in Glyco00 because the pentose phosphate pathway was operational.

Several hypothesis can be drawn to optimize the carbon flux toward GA production with additional metabolic engineering. According to our model, the glucose-6P dehydrogenase encoded by *zwf*, which catalyzes the entrance of D-glucose into the pentose phosphate pathway should be deleted, as well as of *eda-edd* of the Entner-Doudoroff, as both reaction can bypass deletion of *gapA* and generate pyruvate which will then feed the TCA cycle. Additionally, sugar transport can be a valuable target for optimization. Notably, the phosphoenolpyruvate-dependent phosphotransferase (PTS) system does not induce a carbon loss *per se*, but it causes some deviation of PEP that contributes to biomass production. Loss of *PTS* function may have two significant advantages: better assimilate pentose sugars and make possible to uncouple growth and production over time. A bi-phasic process can further be developed to improve the yield. The first phase will be dedicated to the production of biomass from an affordable carbon source in C2, C3, or C4 (ex: malate) to fuel TCA cycle. Meanwhile, the second phase of the fermentation will be dedicated to the production of glycolic acid from lignocellulosic sugars. As highlighted by the flux balance analyses, this second phase could also benefit from further process development, notably by using micro-aerobic conditions to adapt oxidative phosphorylation and redox balance during the production phase. By combining further metabolic engineering and process development, it should be feasible to reach GA production yield close to theoretical maximum yield of 3 moles of GA per mole of C6 and 2.5 moles per mole of C5.

## Conclusion

A new non-natural pathway for GA production has been conceived, implemented and *in vivo* validated. This new pathway termed glycoptimus relies on two pillars. On the one hand, it required the overexpression of three naturally occurring *E. coli* genes, namely *kdsD, fsaA* and *aldA*, whose physiological role are still unclear except for *kdsD*. This synthetic pathway combined with the refactoring of the central carbon metabolism to minimize the carbon loss as CO_2_ at the level of pyruvate should allow to yield theoretically 2,5, and 3 moles of GA from lignocellulosic pentose and hexose, respectively. We successfully demonstrated that this pathway was operational *in vivo*, leading to the production of GA from D-glucose, D-xylose and L-arabinose, albeit at yield that was only at 20–30% of the theoretical ones. Nevertheless, these results argued for a great potential of this microbial process in term of industrial feasibility which will require further optimisation including among others, additional metabolic and strain engineering as well as efficient coupling of NADH reoxidation and energy requirement for sugars uptake and phosphorylation. In addition to ensure stability and robustness of this synthetic pathway, bioprocess-engineering optimisation will be determinant notably because a two-stage process in which a production phase uncoupled from growth is likely the most favorable condition to be conducted to achieve high yield and titer of GA with engineered strains equipped with the glycoptimus pathway.

## Data Availability Statement

The datasets generated for this study are available on request to the corresponding author.

## Author Contributions

CL, CF, and FK performed the experiments. NM carried out the modeling. TW and JF provided guidance for the experimental setups and helped interpreting the results. JF and CL wrote the paper, which has been improved by TW, CF, and NM. All authors approved the final version.

### Conflict of Interest

The authors declare that the research was conducted in the absence of any commercial or financial relationships that could be construed as a potential conflict of interest.
