# Supplementary material for: A New Synthetic Pathway for the Bioproduction of Glycolic Acid From Lignocellulosic Sugars Aimed at Maximal Carbon Conservation
Source: Front Bioeng Biotechnol. 2019 Nov 27;7:359. doi: 10.3389/fbioe.2019.00359 (PMC6900487; doi:10.3389/fbioe.2019.00359)

Supplementary data

A new synthetic pathway for the bioproduction of glycolic acid from lignocellulosic sugars aimed at maximal carbon conservation

Cléa Lachaux[1,2], Cláudio J.R. Frazao[1$], Franziska Krauβer[1], Nicolas Morin[1,2], Thomas Walther[1,2$] and Jean Marie François[1,2]

[1]Toulouse Biotechnology Institute (TBI), Université de Toulouse, CNRS, INRA, INSA, 135 Avenue de Rangueil, F-31077 Toulouse, France

[2]TWB, 3 Rue Ariane, F-31400 Toulouse, France

[§]Present address: TU Dresden, Institute of Natural Materials Technology, 01062 Dresden, Germany

Running title: Synthetic pathway for glycolic acid production

Keywords: Synthetic biology, metabolic engineering, glycolic acid, aldolase, white biotechnology

Table S1: Primers used for PCR amplification of the corresponding genes

Primer	Primer sequence	
CL88	GCCGCGCGGCAGCCATATATGTCGCACGTAGAGTTAC	*kdsD* amplification for cloning in pET28a
CL89	GTCGACGGAGCTCGAATTCGTTACACTACGCCTGCACG	
CL90	GCCGCGCGGCAGCCATATATGGAACTGTATCTGGATACTTC	*fsaA* amplification for cloning in pET28a
CL91	GTCGACGGAGCTCGAATTCGTTAAATCGACGTTCTGCC	
CL92	CTGGTGCCGCGCGGCAGCCATATGTCAGTACCCGTTCAAC	*aldA* amplification for cloning in pET28a
CL92	GTCGACGGAGCTCGAATTCGTTAAGACTGTAAATAAACCACC	
CL22	AGATCCGGCTGCTAACAAAG	Linearization pZ without MCS
CL23	GAATTCTGTGTGAAATTGTTATCC	
CL24	CTTTGTTAGCAGCCGGATCTGTTTAACTTTAAAGGAGGTATAT ATGTCGCACGTAGA	*kdsD* amplification for kdsD-fsaA operon cloning in pZ
CL6	AATAATCCTCCTTTATAGTAATTCTTACACTACGCCTGCACGC	
CL7	GAATTACTATAAAGGAGGATTATTATGGAACTGTATCTGGAT ACTTCAGA	*fsaA* amplification for kdsD-fsaA operon cloning in pZ
CL26	GGATAACAATTTCACACAGAATTCTTAAGACTGTAAATAAAC CACCTGG	
CL29	CTTTGTTAGCAGCCGGATCTGTTTAACTTTAAAGGAGGTATAT ATGTCAGTA	*aldA* amplification for cloning in pZ
CL25	GGATAACAATTTCACACAGAATTCTTAAGACTGTAAATAAAC CACCTGG	
CL126	ATTAAAGAGGAGAAAGGTAC	pZ linearization without Plac
CL125	GTGAAGACGAAAGGGCCTC	
CL127	CGAGGCCCTTTCGTCTTCACCACAGCTAACACCACGTC	proC/D amplification for cloning in pZ
CL128	GTACCTTTCTCCTCTTTAATAAAGTTAAACAAAATTATTTGTA GAGGG	
CL75	AAGGAGGTATATATGTCGCACG	pZA33 linearization without pTac
CL76	GGGCCCTCGTGATACGCC	

Table S2: FBA simulation results for the production of GA via glycoptimus on D-glucose, D-xylose and L-arabinose.

Carbon uptake	$q_{Substrates}$ (mmol.g_{CDW}^{-1}.h^{-1})*		$q_{Products}$ (mmol.g_{CDW}^{-1}.h^{-1})	Production yield ($g_{GA}/g_{Carbon\ Source}$)
	Carbon source	O_2	Glycolate	
D-Glucose	10	15	30	1,27
D-Xylose	10	11	25	1,27
L-Arabinose	10	11	25	1,27

*The carbon substrate flux was arbitrarily set at 10 mmol.g_{CDW}^{-1}.h^{-1}.

Table S3 : Screening strains MG1655 *ΔtktA ΔtktB ΔglcD* used in this project

	**Plasmid for fsaA and kdsD**			**Plasmid for aldA**		
**strain**	**Name**	**Backbone**	**Promoter**	**Name**	**Backbone**	**Promoter**
**Screen01**	pKFA	High copy	Ptac	-	High copy	Ptac
**Screen02**	pKF1	High copy	Ptac	-	-	-
**Screen03**	pKF1	High copy	Ptac	pA1	Medium copy	Ptac
**Screen04**	pKF1	High copy	Ptac	pA4	Low copy	Plac
**Screen05**	pKF1	High copy	Ptac	pA3	Medium copy	Plac
**Screen06**	pKF2	Medium copy	Plac	-	-	-
**Screen07**	pKF2	Medium copy	Plac	pA2	High copy	Ptac
**Screen08**	pKF3	Medium copy	Ptac	-	-	-
**Screen09**	pKF3	Medium copy	Ptac	pA4	Low copy	Plac
**Screen10**	pKF3	Medium copy	Ptac	pA2	High copy	Ptac
**Screen11**	pKF4	Low copy	Plac	-	-	-
**Screen12**	pKF4	Low copy	Plac	pA3	Medium copy	Plac
**Screen13**	pKF4	Low copy	Plac	pA2	High copy	Ptac
**Screen15**	pKF5	Medium copy	proD	-	-	-
**Screen16**	pKF5	Medium copy	proD	pA5	High copy	proD
**Screen17**	pKF5	Medium copy	proD	pA6	High copy	proC
**Screen18**	pKF5	Medium copy	proD	pA7	Low copy	proD
**Screen19**	pKF5	Medium copy	proD	pA8	Low copy	proC
**Screen20**	pFK6	Medium copy	proC	-	-	-
**Screen21**	pFK6	Medium copy	proC	pA5	High copy	proD
**Screen22**	pFK6	Medium copy	proC	pA6	High copy	proC
**Screen23**	pFK6	Medium copy	proC	pA7	Low copy	proD
**Screen24**	pFK6	Medium copy	proC	pA8	Low copy	proC

Figure S1: Carbon fluxes predicted by flux balance analyses for the optimal production of GA from (A) D-glucose, (B) D-xylose, and (C) L-arabinose, using the glycoptimus pathway. Flux values are normalized against a carbon source uptake flux of 1.

Figure S2: Determination of Gibbs energy ($\Delta rG'^0$) of each enzymatic reaction leading from D-glucose or pentose sugars to GA by the glycoptimus pathway. The $\Delta rG'^0$ was calculated using the eEquilibrator tool (http://equilibrator.weizmann.ac.il/). Abbreviation: glk : glucokinase, pgi: phosphoglucoseisomase; tkt: tranketolase; xylA: xylulose isomerase; xylB: xylulokinase; rpe: ribulose-5-phosphate-3-epimerase; araA: arabinose isomerase; araB: ribulokinase; araD: L-ribulose-5-phosphate-4-epimerase, kdsD: D-arabinose-5-phosphate aldolase; aldA: glycoaldehyde dehydrogenase.

Figure S3: Scheme of the screening method to investigate *in vivo* functioning of the glycoptimus pathway. The *E.coli* strain used was MG1655 *ΔtktA ΔtktB ΔglcD* defective in transketolases and glycoladehyde dehydrogenase (*glcD*)

Figure S4 : MG1655 *ΔtktA ΔtktB ΔglcD* strain transformed with various combination of expression systems of the glycoptimus pathway. Twenty-four different expression systems were constructed (see Table S3 for description of plasmid) resulting from a combination of *kdsD-fsaA* operon with *aldA* under inducible or constitutive transformed and carried by low, medium or high copy vector.

Figure S5: Relationship between GA production, D-xylose consumption and D-xylulose excretion in MG1655 ΔtktA ΔtktB ΔglcD screening strains. The transformants were initially cultivated for 16 -24 24 h in M9 containing 0.1% LB. Then, cells were collected (centrifugation at 4 000 rpm for 5 min at RT), washed once with water and re-suspensed at DO_{600} of 0.5 in 50 mL of the same medium in 250 ml baffled shake flask. Growth was monitored at 600 nm and when OD_{600} reached a stable value, sample was taken to measure GA, D-xylose and D-xylulose in the supernatant. Results shown are the mean of two independent experiments.

Figure S6: Activity of FsaA on D-xylulose. Assay was carried out as in Material & Methods using purified FsaA enzyme.

Figure S7: Genetic targets for metabolic engineering of *E. coli* to favour carbon flux into GA production. The genetic targets to be deleted are highlighted in red with a red cross at the reaction step. In blue are indicated the

genes of the glycoptimus module, which were carried out on plasmids pKF6 for *kdsD-fsaA* and pA7 for *aldA*. In orange is represented *galP* expressed under the strong proD promoter.

Figure S1

(A)

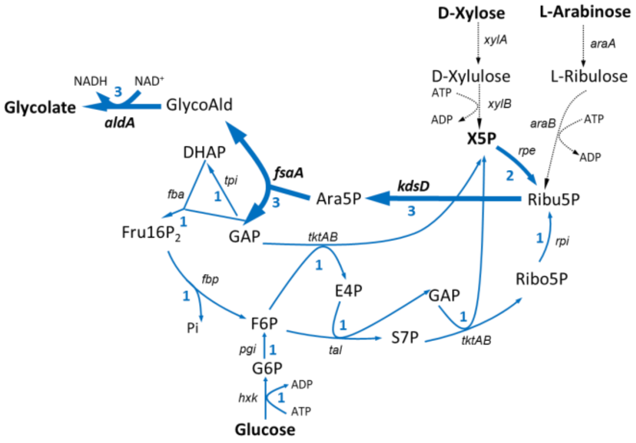

D-Glucose + ATP + 3 NAD^{+} → 3 Glycolate + ADP + P_i + 3 NADH

(B)

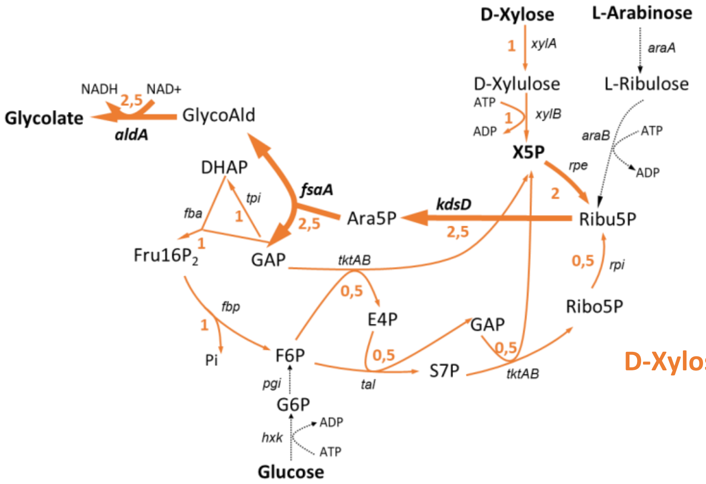

D-Xylose + ATP + 2,5 NAD^{+} → 2,5 Glycolate + ADP + P_i + 2,5 NADH

(C)

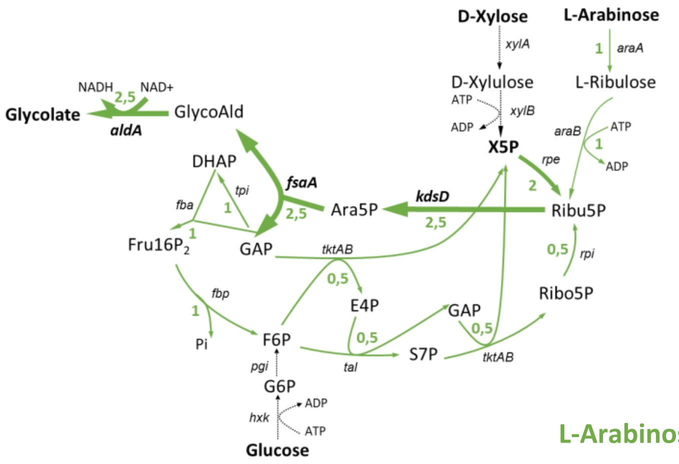

L-Arabinose + ATP + 2,5 NAD^{+} → 2,5 Glycolate + ADP + P_i + 2,5 NADH

Figure S2 (Lachaux et al.)

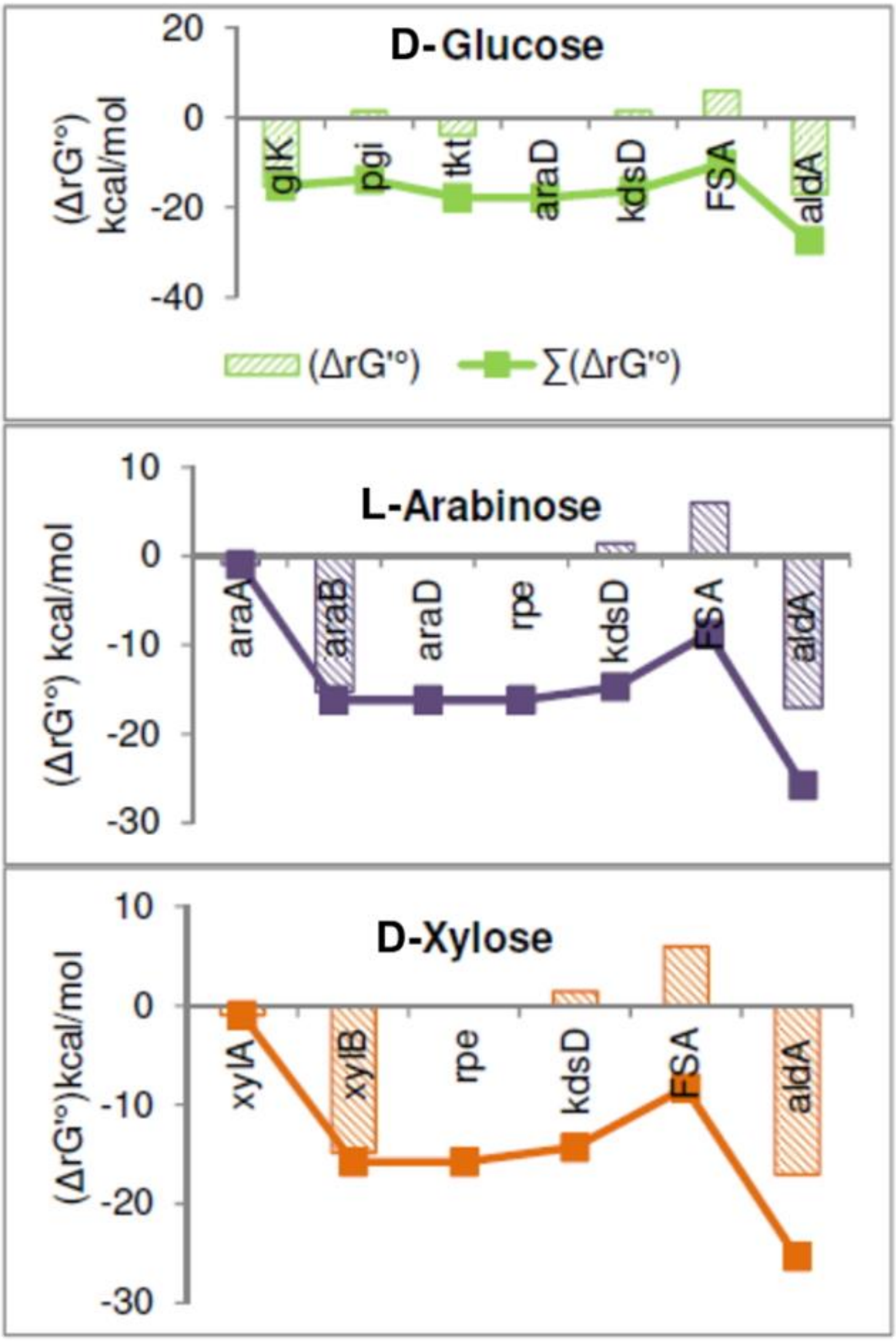

Figure S3 (Lachaux et al.)

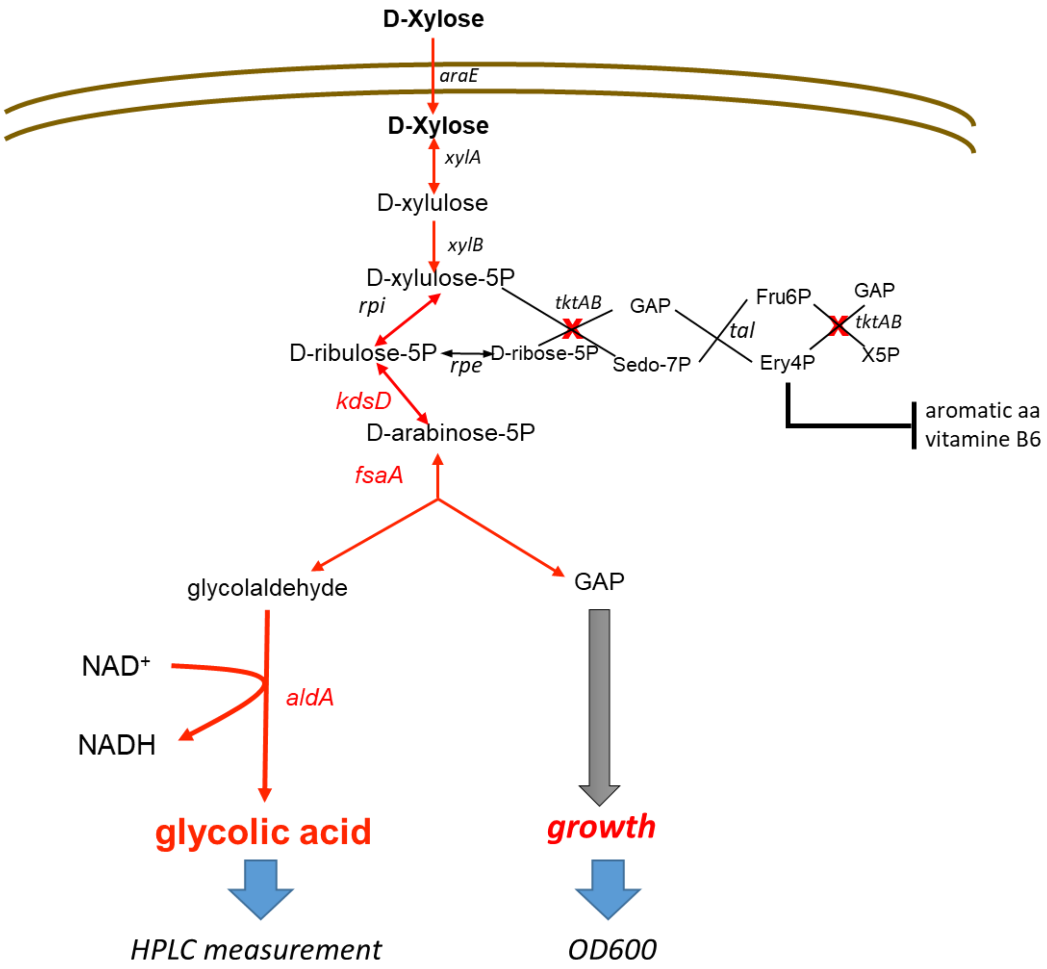

Figure S4 (Lachaux et al.)

PLASMID FOR *fsaA* AND *kdsD* (pKF)

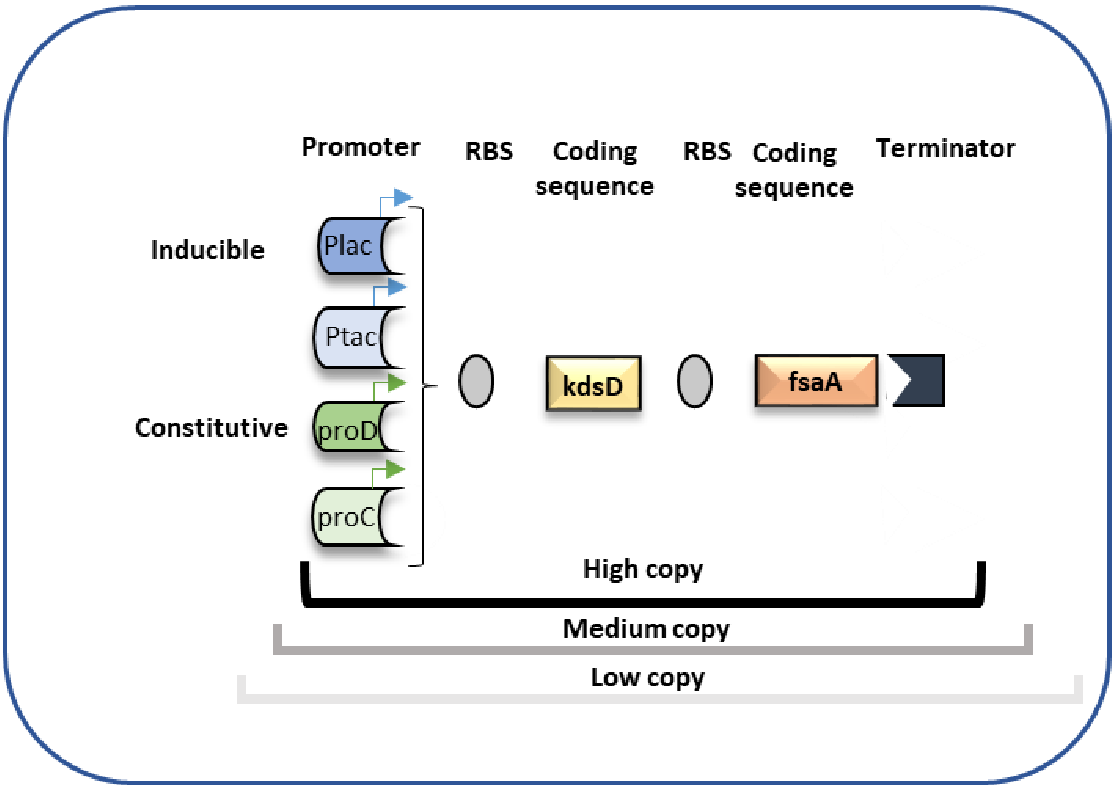

PLASMID FOR *aldA* (pA)

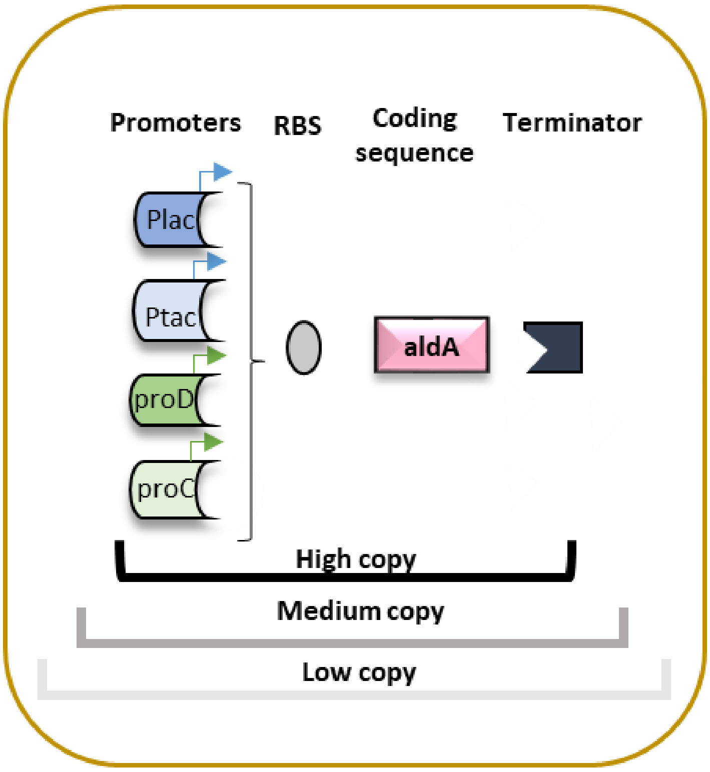

Figure S5 (Lachaux et al.)

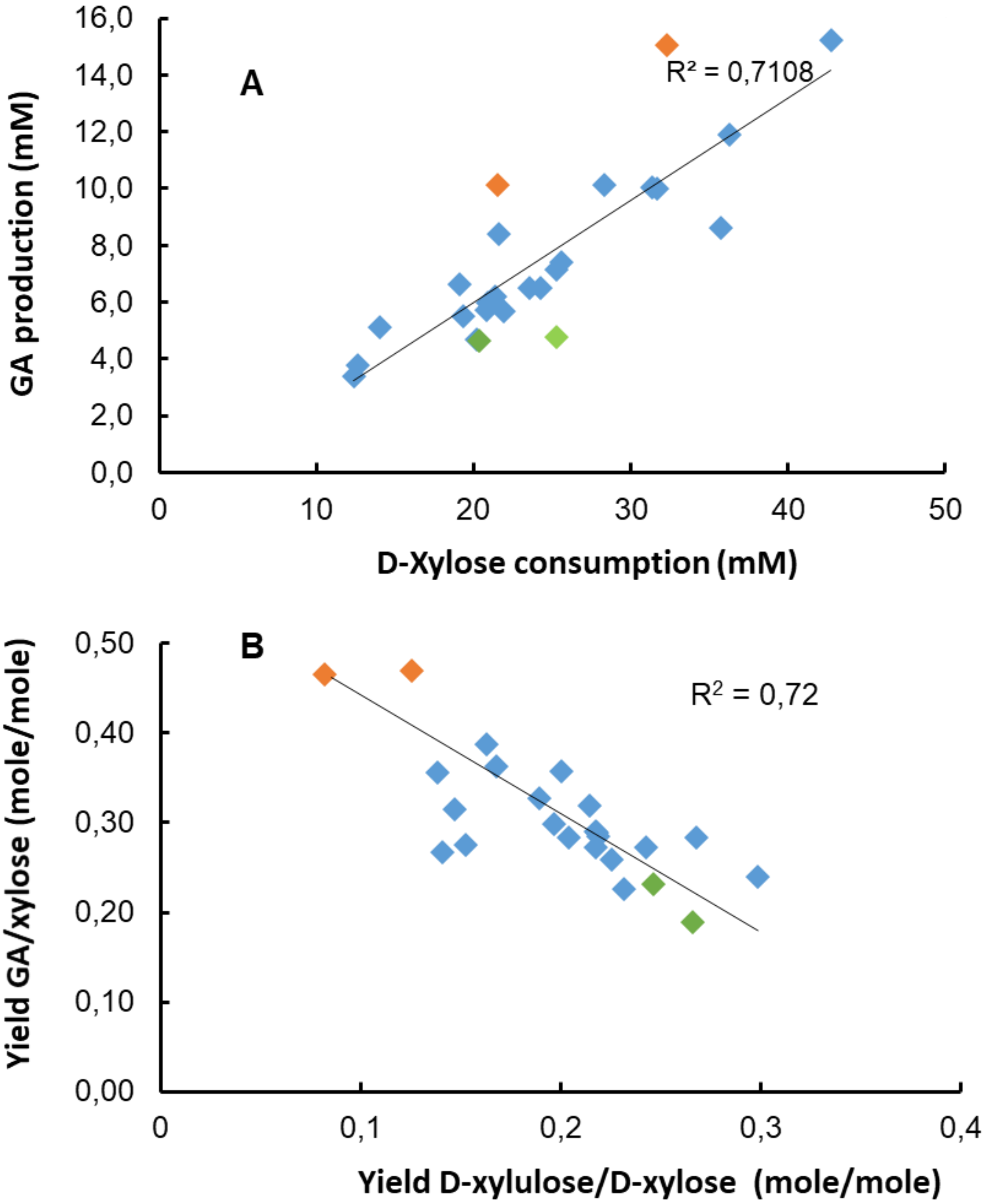

Figure S6 (Lachaux et al.)

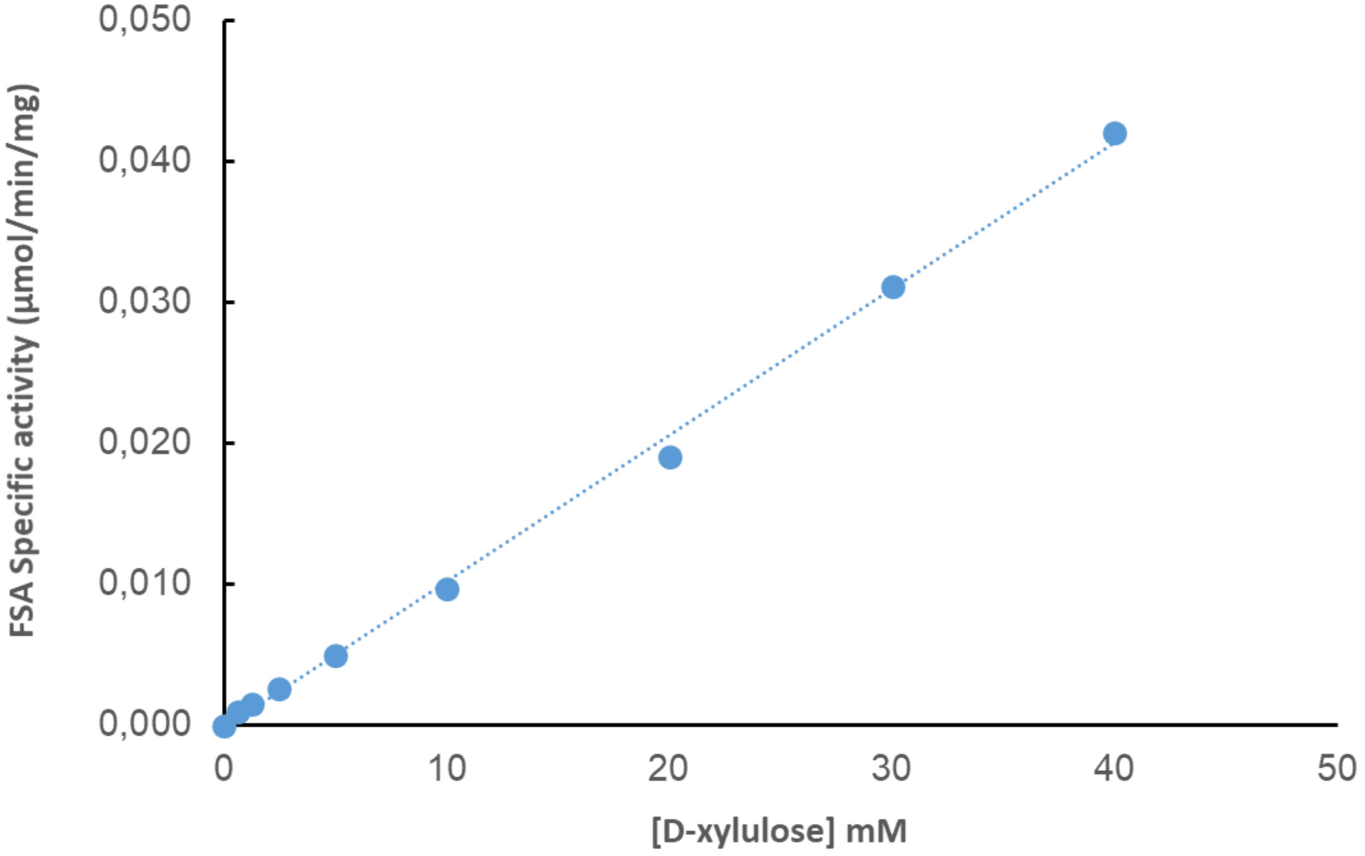

Figure S7 (Lachaux et al.)

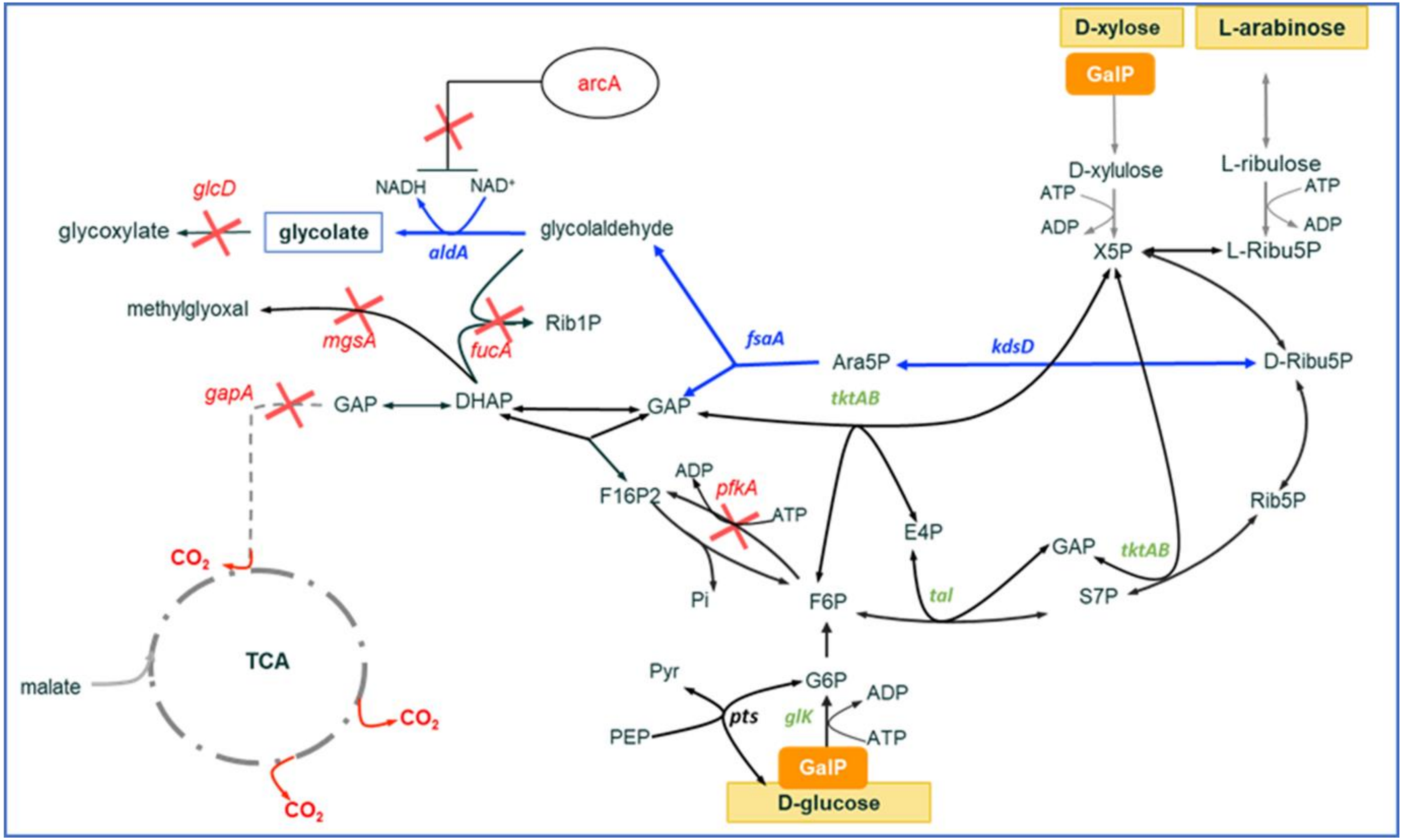

Supplement: Supplementary file 1 [file Data_Sheet_1.PDF]
